# Strongly Modulated Friction of a Film-Terminated Ridge-Channel Structure

**DOI:** 10.1038/srep26867

**Published:** 2016-05-26

**Authors:** Zhenping He, Chung-Yuen Hui, Benjamin Levrard, Ying Bai, Anand Jagota

**Affiliations:** 1 Department of Chemical & Biomolecular Engineering, Lehigh University, Bethlehem, PA, USA; 2 Department of Mechanical & Aerospace Engineering, Cornell University, Ithaca, New York, USA; 3 Michelin Corporation, European Center of Technologies, rue bleue, ZI Ladoux, 63112 Clermont-Ferrand, France; 4 Bioengineering Program, Lehigh University, Bethlehem, PA, USA.

## Abstract

Natural contacting surfaces have remarkable surface mechanical properties, which has led to the development of bioinspired surface structures using rubbery materials with strongly enhanced adhesion and static friction. However, sliding friction of structured rubbery surfaces is almost always significantly lower than that of a flat control, often due to significant loss of contact. Here we show that a film-terminated ridge-channel structure can strongly enhance sliding friction. We show that with properly chosen materials and geometrical parameters the near surface structure undergoes mechanical instabilities along with complex folding and sliding of internal interfaces, which is responsible for the enhancement of sliding friction. Because this structure shows no enhancement of adhesion under normal indentation by a sphere, it breaks the connection between energy loss during normal and shear loading. This makes it potentially interesting in many applications, for instance in tires, where one wishes to minimize rolling resistance (normal loading) while maximizing sliding friction (shear loading).

Friction arises whenever two materials in contact move relative to each other, and its deliberate control is of great practical importance in many applications, e.g., tires^1^, windshield wipers and seals^2^, and for locomotion in robots^3^. Inspired by biological attachment systems^4^,^5^,^6^, several recent studies have shown that some contact mechanical properties, such as adhesion, can be strongly enhanced, significantly attenuated, or made switchable by appropriate design of near-surface architecture^7^,^8^,^9^,^10^,^11^,^12^. Many of the bio-mimicked and bio-inspired structures^7^ share the features of a fibrillar surface terminated by a “mushroom”-like structure^13^,^14^,^15^,^16^, a compliant thin film^17^, or use sub-surface microstructures^18^. Adhesion enhancement often results in increased static friction. Fibrillar surfaces often exhibit smooth and stable sliding with much lower friction force compared to a control flat surface^19^,^20^. Surfaces patterned with mushroom-ended micro-pillars^21^ have enhanced static friction. However, sliding friction is typically lower than for a flat control. A film-terminated fibrillar microstructure strongly enhances static friction^22^,^23^ but sliding friction shows no enhancement compared to a flat control. Similarly, surface wrinkles also reduce sliding friction^24^. Thus, structured surfaces generally reduce sliding friction and therefore it is of interest to look for mechanisms and architectures that enhance it.

In the work presented here we studied the properties of elastomeric samples with an anisotropic surface structure comprising a periodic array of parallel ridges and channels covered by a thin film of the same material (Fig. 1). As described in *Methods*, we measured frictional force by sliding the sample relative to a stiff spherical indenter. We likewise measured adhesion by indentation using a glass sphere.

Figure 2 shows results of friction experiments on a set of samples with ridge height *D* of 40 *μm*, normal load of 1 mN, film thickness of 10 *μm*, and varying spacing *S* (20–125 *μm*), when the direction of motion is orthogonal to the ridges. For the two samples with largest spacing, D40S125 and D40S110, sliding friction (>45 mN) is strongly enhanced by more than a factor of three compared to the flat control (*f*_*c*_ ~12 mN). With further reduction in spacing *S* there is a sharp drop in friction (D40S95) to roughly the value for the flat control. Spacing smaller than 95 μm results in significantly lower friction (~3 mN for D40S50). Thus sliding friction can vary from ¼ to 3 times *f*_*c*_ depending on spacing, and the transition from friction reduction to enhancement is quite abrupt (Fig. 2B). (See SI.2 for data under a broad range of conditions.)

Figure 2(C) shows images of the contact region for sample D40S65 before (C1) and during sliding (C2). (See SI for movie D40S65_move_Speed5_10g_1.mp4 and Fig. S8 for the flat control). Ridges within the contact region bend considerably, and the overall contact region expands due to the attendant increase in compliance. However, bending breaks contact between the terminal film and indenter surface, resulting in significant reduction in friction. Figure 2(D) shows a sequence of images of the contact region for sample S125 along with sketches of the cross-section to illustrate the deformation mode. As the indenter imposes shear on the sample, ridges are stretched and bent, and the contact between indenter and roof film at the contact edge is partially lost (Fig. 2). Until this point, the deformation is qualitatively similar to that seen in Fig. 2 for the sample with smaller spacing. However, with continued shear, the bending and stretching of ridges becomes much more severe. The film forms complex folds and gets stretched well beyond the next ridge (Fig. 2).

In all cases, force measurements vary periodically with sliding distance. For samples with spacing of 95 μm or less, this period corresponds to the distance between adjacent ridges. However, for the two samples with strongly enhanced sliding friction the distance between two adjacent force peaks is twice the distance between adjacent ridges (See SI Fig. S7 for an FFT analysis of force traces). The oscillation amplitude for samples with friction enhancement is larger compared to the other samples (Detail can be found in S.I. Table S1, and the FFT analysis (Fig. S7) shows consistent results). The formation of inner folds at the leading edge, their propagation through the contact region, and their release at the trailing edge also follows this doubled period. (See videos in SI: D40S125_move_Speed5_10 g_1.mp4, D40S65_move_Speed5_10 g_1.mp4).

FE simulation results (Fig. 3A,B) and cross-sectional optical micrographs (Fig. 3C,D) show the deformation mode more directly. Simulation (Fig. 3A) and experiment (Fig. 3B and video in SI: D40S20_Sideview_Speed5_1.mp4) show interfacial opening and associated reduction in contact area, which is responsible for the significant reduction in friction of samples with small periodic spacing. Figure 3 also shows that if the ridge spacing is smaller than ridge height, deformation of ridges is limited by lateral contact of neighboring ridges, which results in a surface rendered somewhat rough due to deformation (Enlarged detail in the upper right corner of Fig. 3,C3.). The contact opening happening during stable sliding in simulation (Fig. 3A) is consistent with observation in Fig. 3 (the slight difference is due to the different normal load applied to sample). Figure 3B shows FE results for D40S125 captured just before the onset of sliding and folding which corresponds to the video capture Fig. 3. Compare Fig. 3B to Figs 3,D2 and 2,D3. There is significant loss of contact, severe bending of ridges and incipient buckling of the terminal film just as it enters the contact. (See also D40S125_Sideview_Speed5_1.mp4 and D40S125_Sideview_Speed5_2.mp4.)

Figures 2 and 3 establish that for samples with ridge spacing smaller than 80 μm the significant bending of the ridges is accompanied by loss of contact, resulting in reduction of friction. We emphasize that such reduction in friction is the norm for structured elastomeric surfaces. However, for sufficiently large separation between ridges, there is a transition to a more complex mode of deformation involving buckling of the terminal film and formation of multiple internal folds (Figs 2 and 3), which is accompanied by a strong increase in sliding friction. The ridges are stretched from their original length of 40 μm to about 70 μm during sliding while the film is stretched to 3 times its original length and folded (Fig. 2). Three sequential video frames near release of the contact (Fig. S9) illustrate a mechanical instability in which the roof film and ridges release elastic energy, which we presume is lost.

The anisotropic nature of the structures results in very different behavior when measuring friction along the ridges. Figure 4A shows how sliding friction varies with ridge spacing at normal load of 1 mN. There is a slight but systematic decrease with increasing spacing, except for the two largest spacings, for which the sliding friction can be quite large albeit with significant variability. (See also SI.2). The sample with largest spacing, D40S125, shows sliding friction enhancement, some 2.5 times that of the flat control. All samples showed significant random fluctuation of friction force, which is related to formation and abrupt release of small folds resembling Schallamach waves^25^ (Fig. 4B,C and movies D40S125_movealong_Speed5_1.mp4, D40S65_movealong_Speed5_10g_1.mp4). Comparison of videos with the force trace reveals the coincidence of sudden reductions in shear force with change in and abrupt relaxation of wrinkles.

The phenomena described above were substantially preserved under the variations such as normal loads, ridge height, and indenter roughness, illustrating the robustness of the mechanisms just discussed (see SI.2). However, we observed that if the ridge height is too small (10 μm), the film tends to collapse and adhere onto the substrate for larger spacing. In this case the folding mechanism is obviated and friction is uniformly reduced significantly due to the decreased contact area.

Previous experiments on the related film-terminated *fibrillar* structure showed strong enhancement of *static* friction caused by the same crack-trapping mechanism that results in strongly enhanced adhesion. In contrast, the film-terminated *ridge/channel* structure studied here shows strong enhancement of sliding friction. Consistent with this connection between static friction and adhesion, we found that the crack-trapping mechanism is inoperative in the film-terminated ridge/channel structure and its adhesion to a flat surface as measured by indentation is not significantly different from that of a flat control.

For friction orthogonal to the ridge direction, we ask: (a) What are the conditions for transition from significantly reduced to strongly enhanced sliding friction? (b) When sliding friction is strongly enhanced, what are the associated mechanisms? Figs 2C and 3C show that the condition spacing *S* > *D* is necessary for enhanced sliding friction because otherwise the ridges make lateral contact with each other, obviating the possibility of buckling and folding of the terminal film. (Note that the minimum friction in Fig. 2A (inset) is for D40S50, for which spacing approximately equals the ridge height.) However, there is a limit on how wide spacing can be made before the terminal film collapses and sticks to the substrate below it (e.g., sample set D10 shows no friction enhancement). In SI.5 we show that the following condition must be satisfied to avoid film collapse: 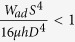. Finally, we propose a third necessary condition for transition to internal folding and high friction, which is that the terminal film just entering the contact region must buckle under the compressive load transmitted to it (Fig. 3). In SI.6 we provide a simple model that quantifies this condition, with the result that 
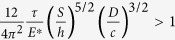
, where *τ* is the average frictional stress in the contact region and *c* is ridge width. For our parameters, this predicts that a periodic spacing of at least 50 microns is necessary.

To address question (b) we first note that the reduction in friction for samples with spacing (smaller than 80 μm) is readily understood as resulting from loss of contact associated with the bending of the ridges. The shape of the joint between the ridge and the film is critically important. Specifically, when the top of the ridge meets the film at a sharp right angle, as is the case when the film is partially pre-cured, the ridge bends relatively easily, resulting in opening of the interface. If we estimate the reduction in contact area using the finite element results for the corresponding cases, we find that the actual area of contact for the flat control, D40S20, and D40S50 is in the ratio 3.4:1.3:1. This is consistent with the ratio of measured sliding friction of these samples, which is about 3.5:1.7:1.

To understand the mechanisms responsible for the strongly enhanced sliding friction in samples with large spacing, we focus on D40S125, which has an enhancement ratio between 3 and 4. Careful analysis of videos for this sample (D40S125_move_Speed5_10 g_1.mp4, D40S125_move_Speed5_10 g_2.mp4) suggests the following possible contributions to energy loss (in addition to the sliding friction of the indenter against the top surface):

(1) Loss of elastic energy stored in bending and stretching the ridges,

(2) Loss of elastic energy stored in stretching the terminal film,

(3) Frictional losses due to relative sliding at internal folded interfaces,

(4) Energy loss due to adhesion hysteresis associated with closing and opening of internal interfaces.

To quantify the potential contribution of each of these mechanisms, we estimate the energy lost in transferring a slice of the material from the leading to the trailing edge. The arguments are developed in more detail in SI.4; here we present the conclusions.

We find the contribution to energy loss of mechanisms 1 & 4 to be negligible in comparison to the overall energy loss. The strong periodic undulation in friction force supports the hypothesis that elastic energy stored in the deformation is released unstably and hence largely lost. We find that loss of energy stored in the more substantial stretching of the membrane contributes significantly, accounting for about 25% of the overall frictional loss. Folding of the structure causes the formation of several internal interfaces that slide relative to each other sufficiently to increase frictional losses by a factor of 3–4.

We conclude, therefore, that the transition to strongly enhanced friction is governed by a buckling phenomenon which leads to the formation of multiple internal folds. The formation and release of these structures causes significant internal sliding and unstable release of stored elastic energy. Together these account for the strong increase in sliding friction.

The frictional response for motion along the ridges is quite different. It is accompanied by buckling of the film near the leading edge of the indenter. For most of our samples the post-buckled state of the terminal film is retained as it enters under the indenter and is released at the trailing region of contact. However, for the largest spacing samples, after buckling the terminal film appears to kink, fold, and adhere to itself. This change in deformation mode is accompanied by a significant increase in static friction.

## Methods

The samples were fabricated using poly(dimethylsiloxane) (PDMS, Sylgard 184, Dow Corning) following the procedure of^22^. Briefly, an array of periodic ridges is created by molding PDMS into a micro-fabricated silicon template. After being peeled off the Si template, the PDMS substrate patterned with ridges is placed on a partially pre-cured PDMS film with thickness controlled through spin coating. The assembly is then cured at 80 °C for 2 hours. The samples studied here are of different ridge heights *D*: 10, 30 and 40 μm, film thickness *h* of about 10 μm and substrate thickness of about 700 μm. The periodic spacing between adjacent ridge centers is varied from 20 to 125 μm. A PDMS flat control sample was also fabricated under the same conditions.

To measure friction under controlled normal load, we use the same apparatus and procedure as in^22^ (SI.1, Fig. S1(A)). Briefly, a glass indenter 8 mm in radius and coated with a self-assembled monolayer, is brought into contact with a sample (supported on a glass slide) that is placed on an inverted microscope under normal load controlled by a mechanical balance (Ohaus 310D). Then, with the indenter fixed, the sample is driven by a variable speed motor (Newport ESP MFA-CC) at a constant speed of 5 μm/sec. The frictional force is recorded by a load cell (Honeywell Precision Miniature Load Cell) while the deformation mechanism is visualized and recorded through an inverted microscope. Samples are moved in two distinct directions: along (parallel to) and orthogonal to the ridges/channels. Measurements were performed for three sets of samples with different ridge heights: 10, 30 and 40 μm. Samples in each set have different periodic spacing between adjacent ridge centers of 20, 35, 50, 65, 80, 95, 110 and 125 μm. For each sample, we applied different normal loads and measurements were repeated at least twice. Selected results are presented in the main manuscript; see SI.2 for more results.

To better understand the deformation mechanism, friction experiments were also carried out with the sample rotated by 90 degrees, exposing a cross-sectional view of the samples. The glass slide supporting the PDMS samples was placed vertically with the sample cross-section facing the microscope. Then, the spherical glass indenter was brought into contact with the sample surface at the same horizontal level as sample’s cross section surface. By recording the video signal while moving the samples relative to the glass indenter we were able better to observe and understand the deformation modes (SI, Fig. S1(B)) but with lack of control on the normal force. Indentation experiments were also carried out to measure adhesion using the same experimental set-up as in^22^.

We conducted 2D (plane strain) finite element simulations of a cylindrical indenter in contact with and sliding on a structured surface to analyze and interpret the phenomena observed during friction experiments. We used the commercial finite element program, ABAQUS^®^, augmented with custom-written cohesive interface elements^26^. The simulation is carried out in two steps. In the first, the rigid indenter is brought into contact with the surface of the sample and in the second it is moved along its surface. In addition to elastic properties of the sample, we prescribe a constant frictional stress. Further details about the simulations can be found in SI.3.

## Additional Information

**How to cite this article**: He, Z. *et al.* Strongly Modulated Friction of a Film-Terminated Ridge-Channel Structure. *Sci. Rep.* **6**, 26867; doi: 10.1038/srep26867 (2016).

## Supplementary Material

Supplementary Information

Supplementary video

Supplementary video

Supplementary video

Supplementary video

Supplementary video

Supplementary video

Supplementary video

Supplementary video

## Figures and Tables

**Figure 1:**
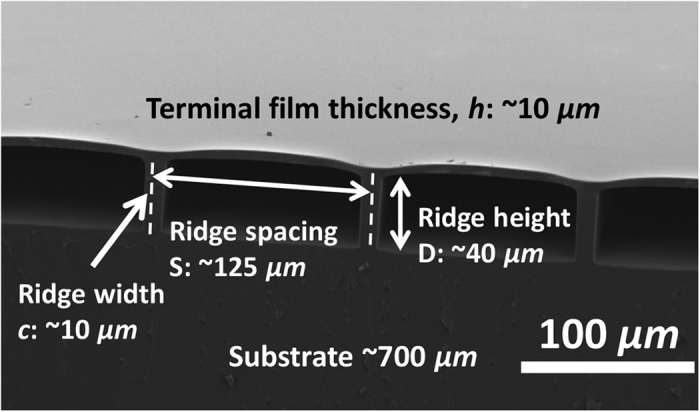
Scanning electron micrograph of a typical sample showing an array of ridges and channels on the surface of a relatively thick substrate, capped by a terminal film. We have studied samples with three different ridge heights D (10, 30 and 40 *μm*), fixed ridge width *c* (10 *μm*), several values of spacing between adjacent ridge centers S (20 to 125 *μm*) and fixed film thickness, *h* (~10 *μm*). Samples are denoted as DxxSyyy, where “xx” provides ridge height and “yyy” provides spacing between ridge centers.

**Figure 2:**
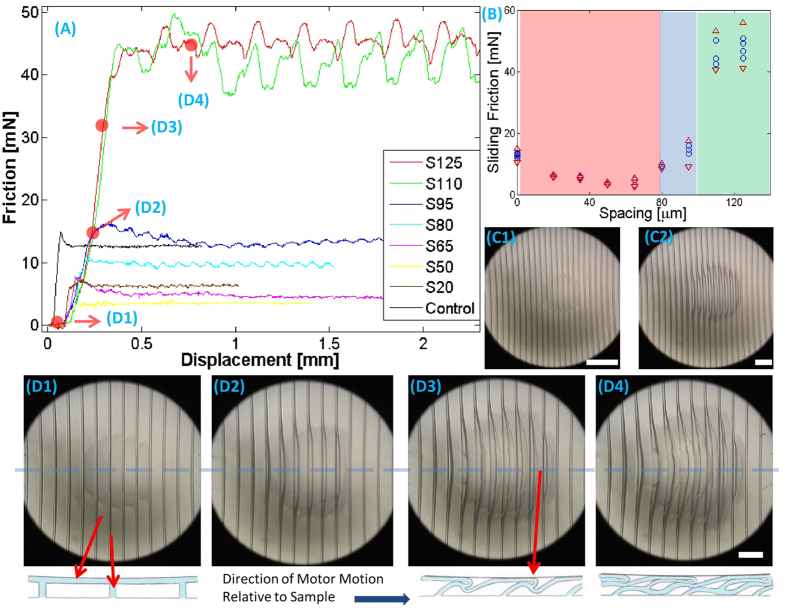
Friction of film-terminated ridge-channel samples orthogonal to the ridge direction. (**A**) Force resisting sliding of an indenter against an unstructured flat control and film-terminated ridge-channel samples with varying spacing at relative speed of 5 *μm*/sec. (**B**) Friction force for different experimental trials is shown by the circles. The range of variation, i.e., the minimum and maximum friction force measured, is shown by the red triangles. (The flat control is assigned a ridge spacing value of 0.) (**C**) Image of contact region for sample S65 before sliding (C1) and during sliding (C2). Bending of the ridges inside the contact region is considerable and the contact region expands due to the attendant increase in compliance. Careful examination of the micrographs reveals significant loss of contact between the terminal film and indenter surface. (**D**) Sequence of images of the contact region for sample D40S125 along with sketches of the cross-section depicting the deformation mode (See also Fig. 3). D1 shows the contact region before sliding, D2 and D3 show intermediate stages while D4 shows the contact region during steady sliding. The white scale bars (in C1, C2, D4) represents a distance of 200 *μm*. (Since the normal load is 1 mN, the labels on the ‘y’ axis in Fig. 2B can also be read as the friction coefficient.)

**Figure 3:**
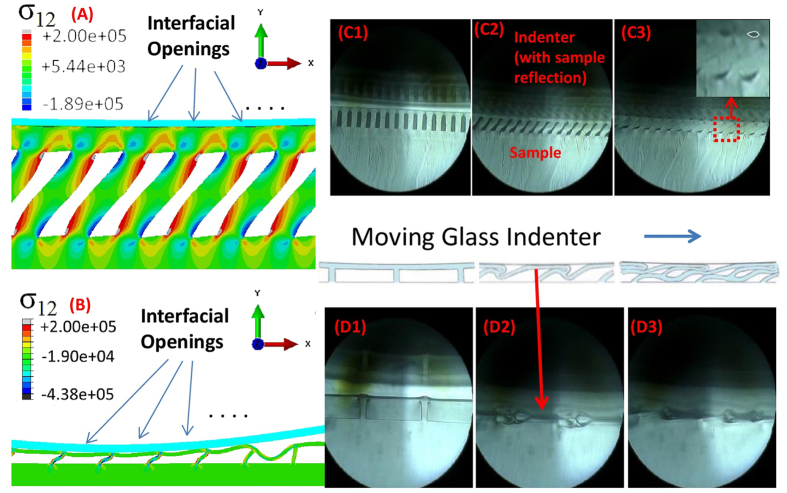
Snapshots from finite element simulations of the deformation for samples D40S20 (**A**) and D40S125 (**B**), where σ_12_ is shear stress in N/m^2^. Fig. 3A is a random capture during stable sliding in simulation, and 3B is the capture just before the onsite of folding. (C1, C2, C3) Side views of sample D40S20 during a friction experiment with progressively increasing shear. C1 corresponds to the state with no motion, C2 to motion of about 20 *μ*m, while C3 is picked randomly during stable sliding. (D1, D2, D3) Side views of sample D40S125 during progressively increasing shear. D1 shows the capture at no motion, D2 is the capture at motion of about 40 *μ*m, while D3 is the one showing serious folding during sliding. The sketches (repeated from Fig. 2) illustrate the deformation mode and correspond to images below them.

**Figure 4:**
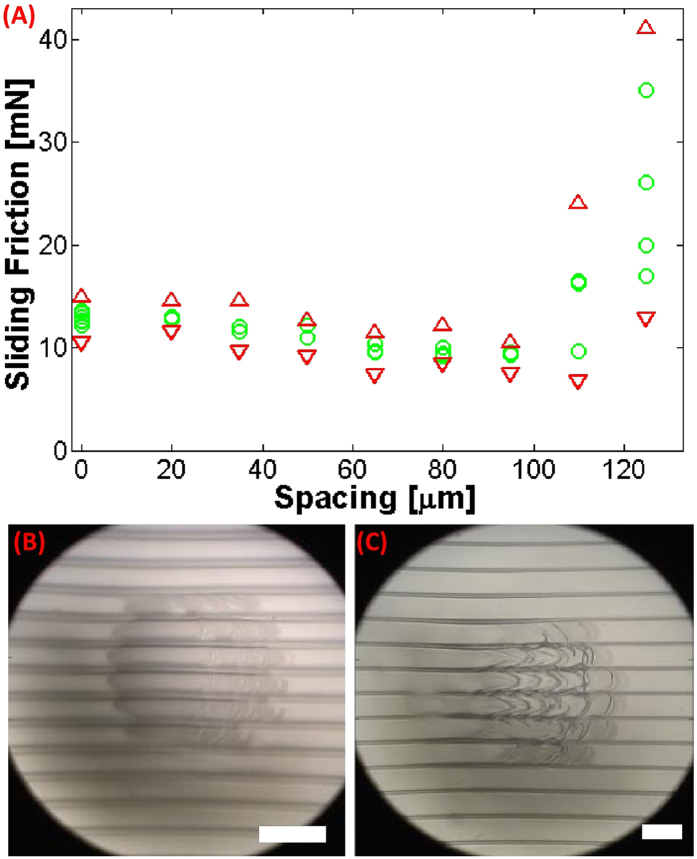
Friction between a spherical indenter and the film-terminated structure for relative motion along the ridges for sample set D40. Fig. 4A shows the sliding friction (green circles) and its range of variation (red triangles). Deformations are visualized through snapshots for sample D40S65 (4B) and sample D40S125 (4C). The scale bar represents a distance of 200 *μm*. (Since the normal load is 1 mN, the labels on the ‘y’ axis in Fig. 2B can also be read as the friction coefficient.)
