# Supplementary material for: Strongly Modulated Friction of a Film-Terminated Ridge-Channel Structure
Source: Sci Rep. 2016 May 26;6:26867. doi: 10.1038/srep26867 (PMC4881016; doi:10.1038/srep26867)
Supplement: Supplementary Information [file srep26867-s1.pdf]

Strongly Modulated Friction of a Film-Terminated Ridge-Channel Structure

Zhenping He[1], Chung-Yuen Hui[2], Benjamin Levrard[3], Ying Bai[1], Anand Jagota[1,4,*]

[1]Department of Chemical & Biomolecular Engineering, [4]Bioengineering Program, Lehigh University

[2]Department of Mechanical & Aerospace Engineering, Cornell University

[3]Michelin Corporation, European Center of Technologies, rue bleue, ZI Ladoux, 63112 Clermont-Ferrand, France

[*] Corresponding Author anj6@lehigh.edu D331 Iacocca Hall, 111 Research Drive, Lehigh University, Bethlehem, PA 18017

Supplementary Information

SI.1. Experimental Set-up

The set-up to measure friction is the same as that described in ref [1], shown in Fig. S1A. Fig. S1B shows the bottom view illustrating the modification of the set-up in Fig. S1A for the experiment in which we visualized deformation modes by viewing the cross-section of the sample. To observe the deformation from this side-view, the glass-slide holding the film-terminated ridge-channel structure is placed vertically to stand on the microscope stage, and indenter is brought horizontally (instead of vertically) into contact with the sample.

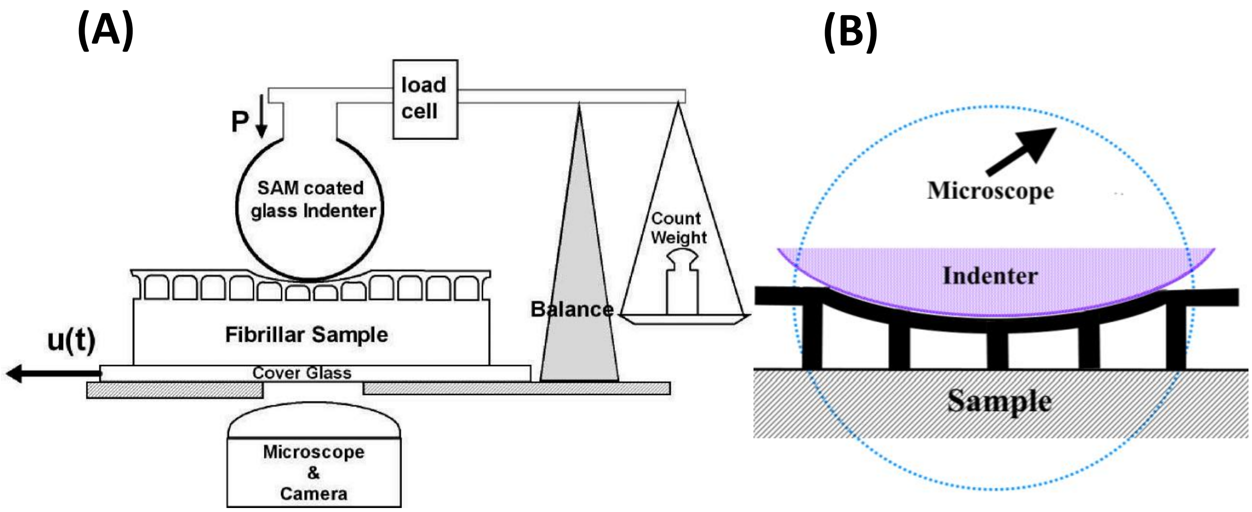

Figure S1. Schematic of experimental set-up for friction measurement (A)(ref [1]). (B) Bottom view of a modified set-up used to visualize deformation modes in cross-section. The dotted circle represents the microscope objective used.

SI.2. Experimental Data for Different Normal Loads and Ridge Heights

The parameters characterizing the anisotropic structures are ridge width (which is fixed at 10 µm for all samples), ridge spacing (distance between two adjacent ridge centers, denoted as S), ridge height (denoted as D) and film thickness *h* (which is controlled between to be ~10 µm). We studied three sample sets of different ridge heights: 10, 30 and 40 µm. For each height we studied ridge spacings of 20, 35, 50, 65, 80, 95, 110 and 125 µm. The substrate thickness was controlled to be about 700µm. The terminal film for each sample was fabricated at the same condition, i.e., same compound recipe, same spin speed, spin time and curing temperature, which ensured that each sample had the same film thickness. Three different normal loads were used (1, 2 and 4 mN). The relative speed between PDMS samples and the glass indenter was constant for all experiment at 5µm/sec.

The shear force vs displacement data for samples D40 at normal load of 1 mN are plotted in Figs. S2 and S3. Fig. S2 corresponds to sliding orthogonal to ridge/channel, while Fig. S3 is for the sliding parallel to (along) the ridge/channel.

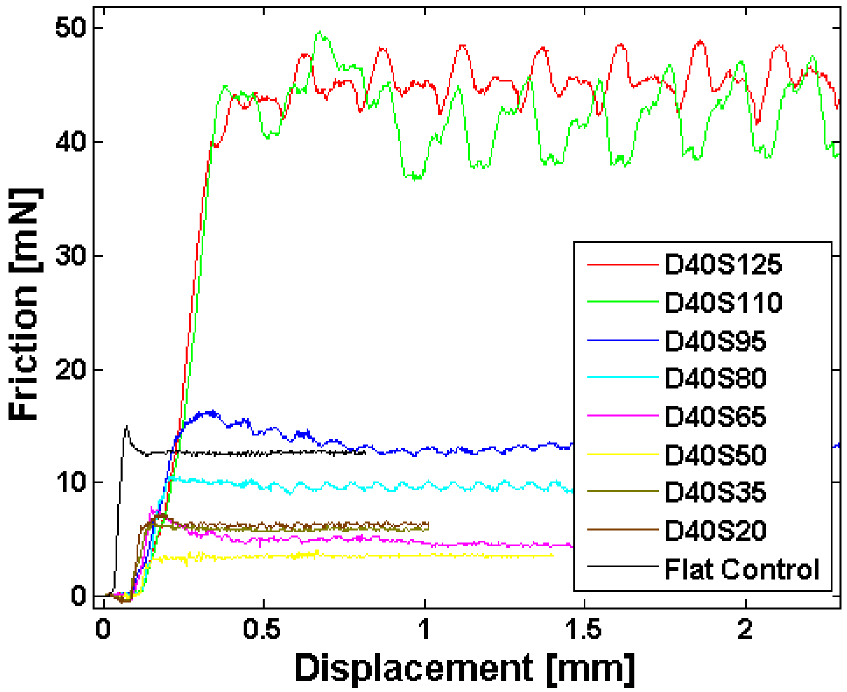

Figure S2. Typical force-displacement trace for film-terminated Ridge/Channel samples with ridge height of 40µm and various spacings with indenter moving orthogonal to ridges at a normal load of 1 mN.

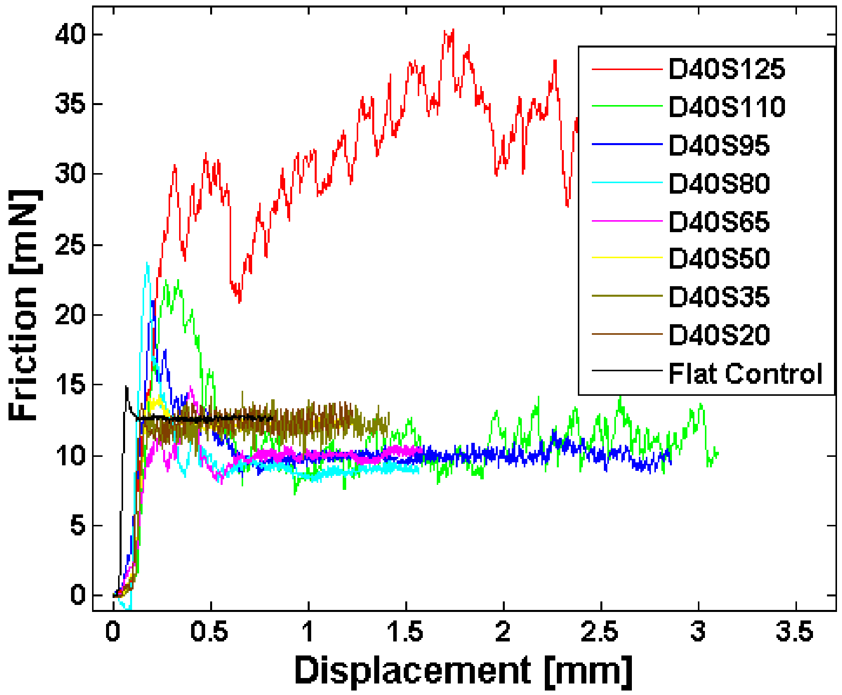

Figure S3. Typical force-displacement trace for film-terminated Ridge/Channel samples with ridge height of 40µm and various spacings with indenter moving along ridges at normal load 1 mN.

When the normal load was increased to 2 mN gram, sample set D40 showed similar shear force vs displacement data. Fig. S4 corresponds to the direction orthogonal to ridge/channel at different normal loads (Color green corresponds to normal load 1 mN, color red corresponds to normal load 2 mN and color blue corresponds to normal load 4 mN). In Fig. S4, circles represent the average friction force value for different measurements while the triangles illustrate the highest and lowest friction force observed in experiments. It is clear that increasing normal load simply scales the overall friction force with no substantial change in the overall trends.

The maximum and minimum friction forces are read based on multiple experiment trials. The average dynamic friction force was calculated based on averaging the oscillating friction force for each experimental trial. The normal load was controlled by the balance as shown in Fig. S1.

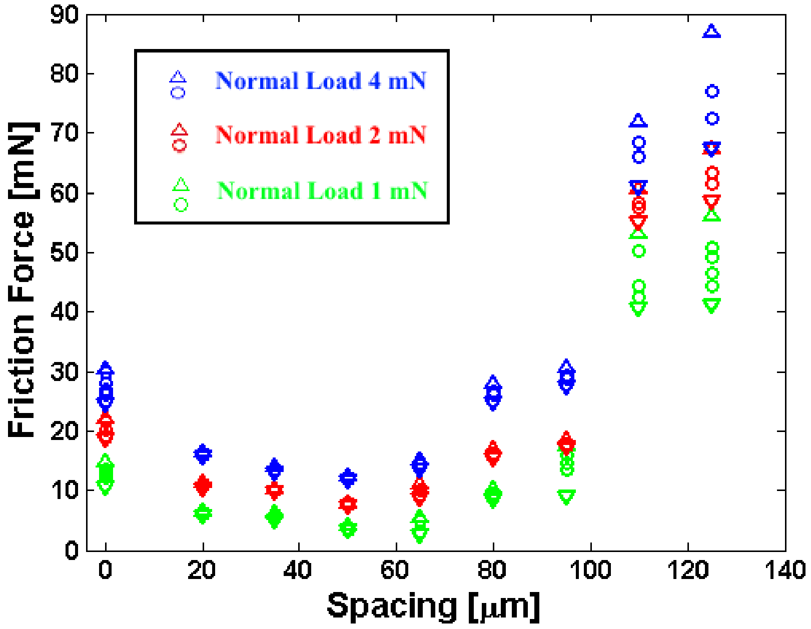

Figure S4. Sliding friction force for film-terminated Ridge/Channel samples with ridge height of 40μm and various spacings with indenter moving orthogonal to ridges at normal load 1 mN (green), 2 mN (red) and 4 mN (blue). The circles represent the average friction force for different experiment trials and the triangles represent the highest and lowest friction values measured.

Due to the strong influence of surface adhesion, for soft matter, friction coefficient is usually not a meaningful parameter and so is not commonly reported. However, the friction coefficient for each sample can be easily calculated by dividing the friction force by normal load. When the normal load is 1 mN, the labels on the 'y' axis can be read as friction coefficient; this also applies to Figs. S4, S5 and S6. The friction force measured at different normal load while moving the indenter along ridges/channels is plotted in Fig. S5. Similar to Fig. S4, different normal loads are separated by the color, and circular and triangular shapes corresponds to the average friction force and ranges respectively. Again, the overall trends are substantially preserved.

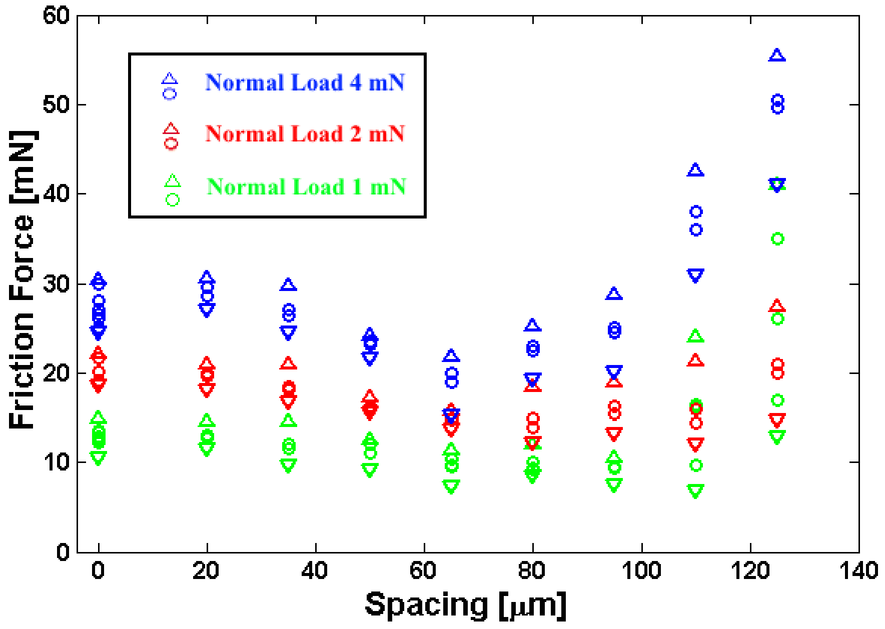

Figure S5. Sliding friction force for film-terminated Ridge/Channel samples with ridge height of 40μm and various spacings with indenter moving along ridges at normal load 1 mN, 2 mN and 4 mN. Circular symbols represent the average friction force for different trials and triangular shapes represent the highest and lowest friction forces in experiments.

Fig. S6A contains the sliding forces at normal load 1 mN (green) and 4 mN (blue) for sample set D30. It shows that sample set D30 has similar trends as set D40. In particular, the enhancement for sample D30S125 is about 3 times at normal load 1 mN and 2 times at higher normal load 4 mN. Interestingly, sample D30S110 does not show friction enhancement at normal load 4 mN compared to the lower normal load 1 mN. This suggests that the critical spacing for transition to the folding mechanism can be modulated by normal load.

Friction for relative motion along ridges/channels, for sample set D30, showed little enhancement except for sample D30S35 (Fig. S6B). All the other samples with different spacings exhibit friction very close to or a bit lower than that of flat control sample.

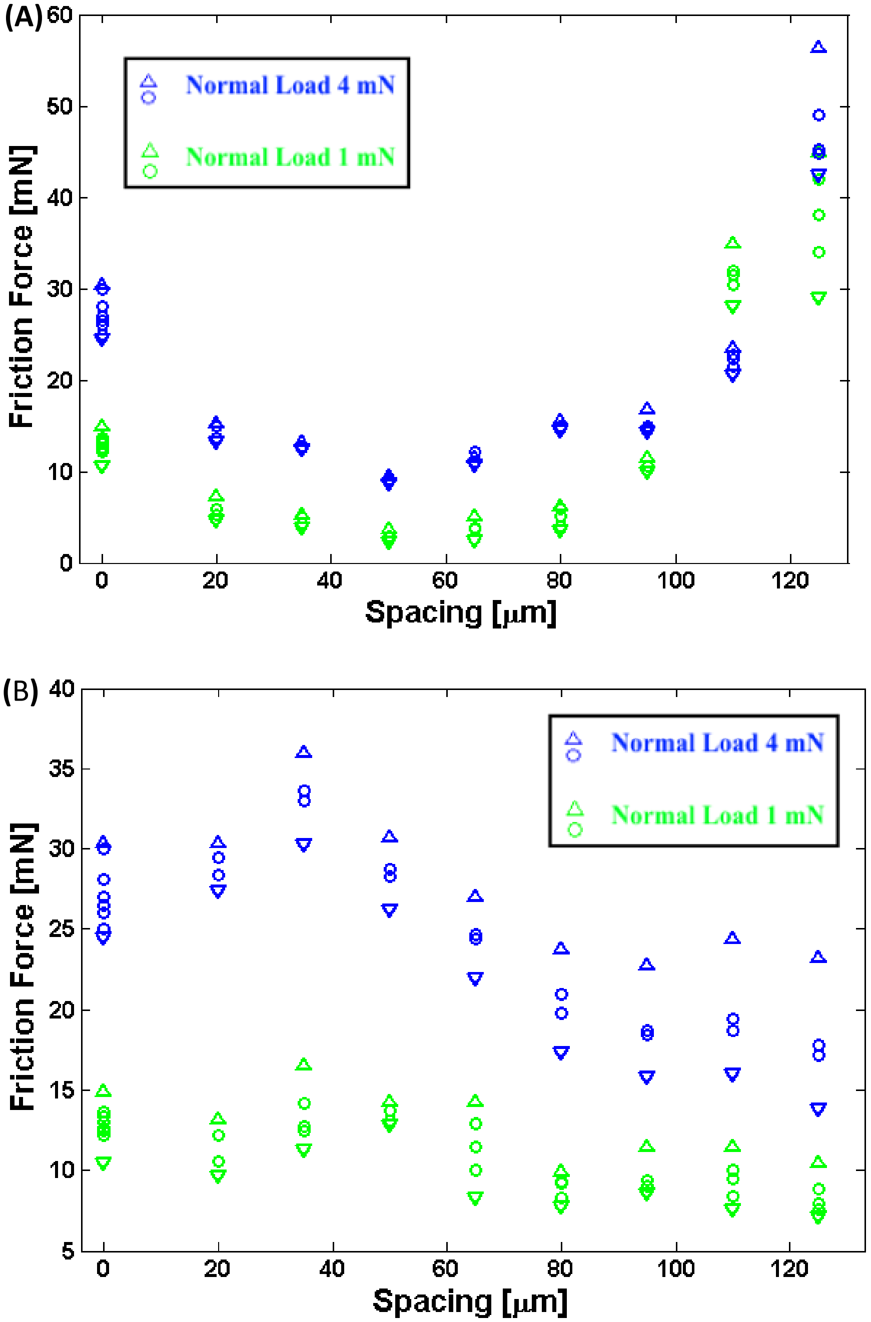

Figure S6. A: Sliding friction force for film-terminated Ridge/Channel samples with ridge height of 30 μm and various spacings with indenter moving orthogonal to ridges at normal load 1 mN (green) and 4 mN (blue). The circular shapes illustrate the average friction force for different experiment trials and the triangles represent the highest and lowest friction value. B: Sliding friction force for film-terminated Ridge/Channel samples with ridge height of 30μm and various spacings with indenter moving along ridges at normal load 1 mN and 4 mN. Circles represent the average friction force for different trials and triangles represent the highest and lowest friction forces in experiments.

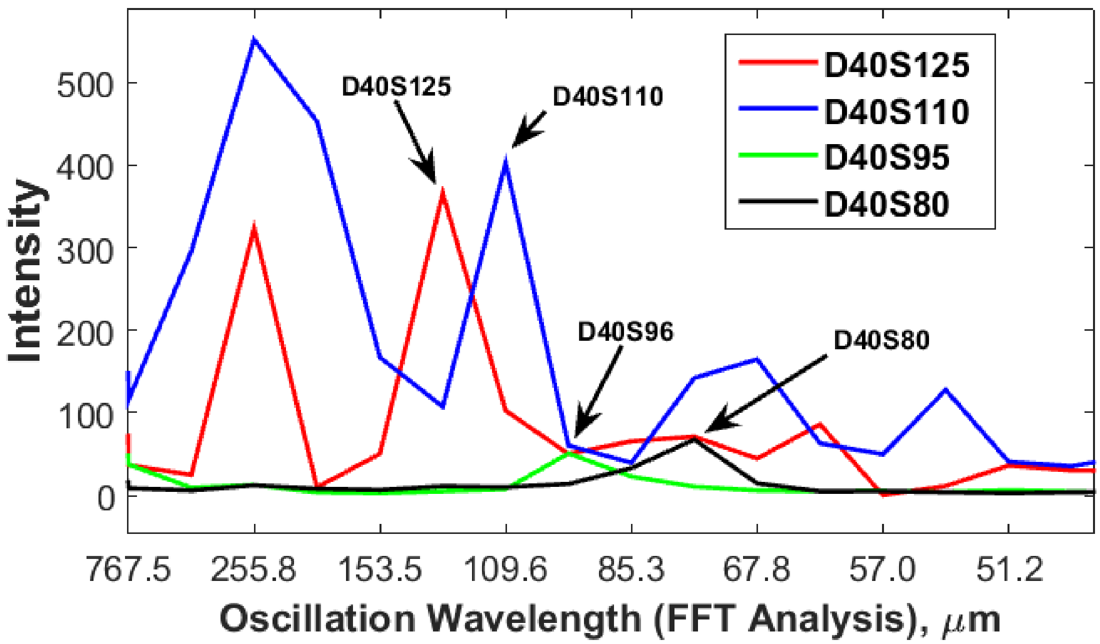

Figure S7. Power spectra of oscillatory friction force for samples (D40S125, D40S110, D40S95 and D40S80).

Due to the periodic structure of our samples, the measured sliding friction is also periodic. Power spectra based on the FFT (Fast Fourier Transform) for samples D40S125 and D40S110 show two main peaks. The first peak corresponds to a wavelength that is twice the minimum ridge-ridge spacing, while the second one corresponds to the ridge spacing. For samples with smaller spacing (D40S95 and D40S80), the spectra corresponds a single dominant period with wavelength the same as the ridge spacing (Fig. S7). FFT analysis also reveals that D40S125 and D40S110 have larger amplitude compared with D40S95 and D40S80. The oscillation amplitude data read from the friction data are provided in Table S1.

Sample	D40S125	D40S110	D40S95	D40S80
Oscillation Amplitude (mN)	2.85	4.1	0.45	0.45

Table S1. Oscillation amplitude of friction measured for samples (D40S125, D40S110, D40S95, D40S80).

Fig. S8 shows the contact region on a flat control sample before and during sliding. The dark region represents the contact region. Fig. S8A shows initial contact before sliding; S9B shows the contact during sliding – the sample moves from left to right against the fixed indenter. In the side-view experiment, we also conducted experiments to follow the deformation as the indenter is pulled away out of contact with the sample. Fig. S9 shows three sequential snapshots capturing

the unsteady release of the folded and stretched state of the terminal film and ridges for sample D40S125. These support the idea that unstable, dynamic, release of stored elastic energy contributes to overall sliding friction.

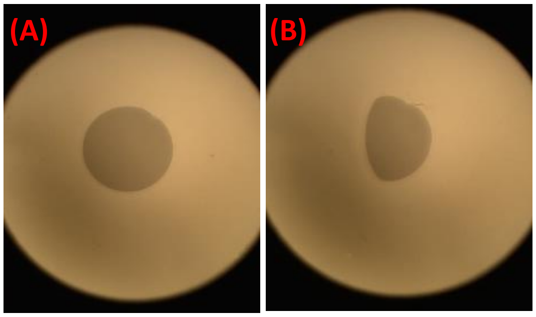

Figure. S8. Screen shots of the contact region change for flat control sample before (A) and during sliding (B).

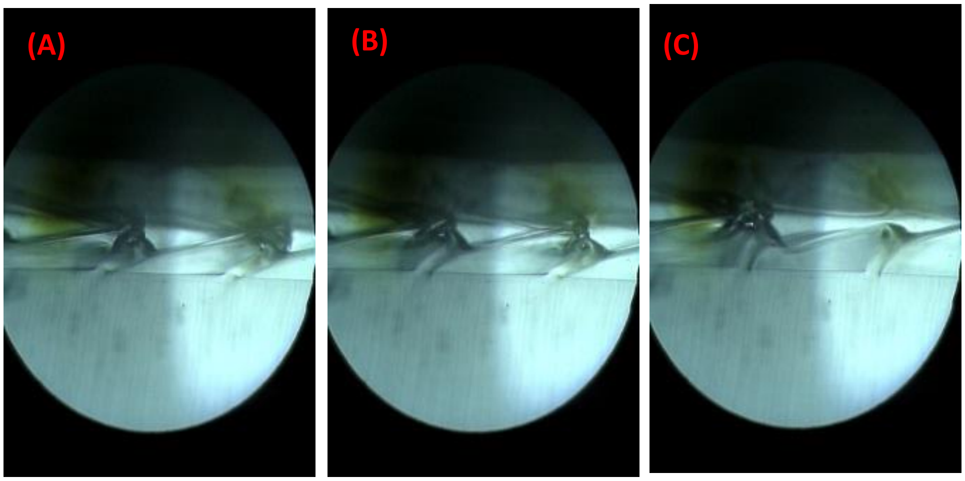

Figure S9. Three sequential screen shots illustrating the unsteady release of folded and stretched structure as the indenter retracts from the sample, D40S125.

SI.3. Finite Element Simulation

The finite element simulations on structured samples included contact and friction but did not account for adhesion. This was because inclusion of cohesive elements to represent adhesion in addition to complexities such as changing contact, friction, and buckling, made the simulations prohibitively slow to converge. We instead accounted approximately for the effect of adhesion by adjusting the normal force in the following manner.

Finite element simulations of contact and sliding of a cylindrical indenter on a flat control sample were performed with and without consideration of the surface adhesion. In this case we were successfully able to conduct simulations using cohesive elements to model adhesion [2]. Comparison shown in these two figures reveals that by appropriately adjusting (increasing) the normal load without adhesion the same friction force (shear tension in Fig. S10) and contact area (Fig. S11) can be obtained as those for the situation where adhesion is considered.

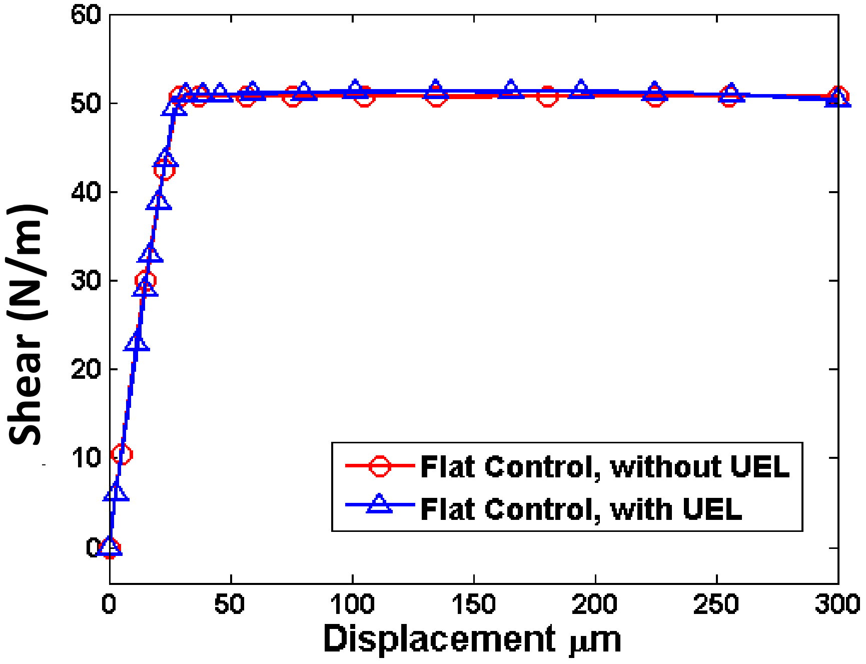

Figure S10. Comparison of plots of shear for flat control based on finite element simulation without and with adhesion (UEL is abbreviation for user element for adhesion).

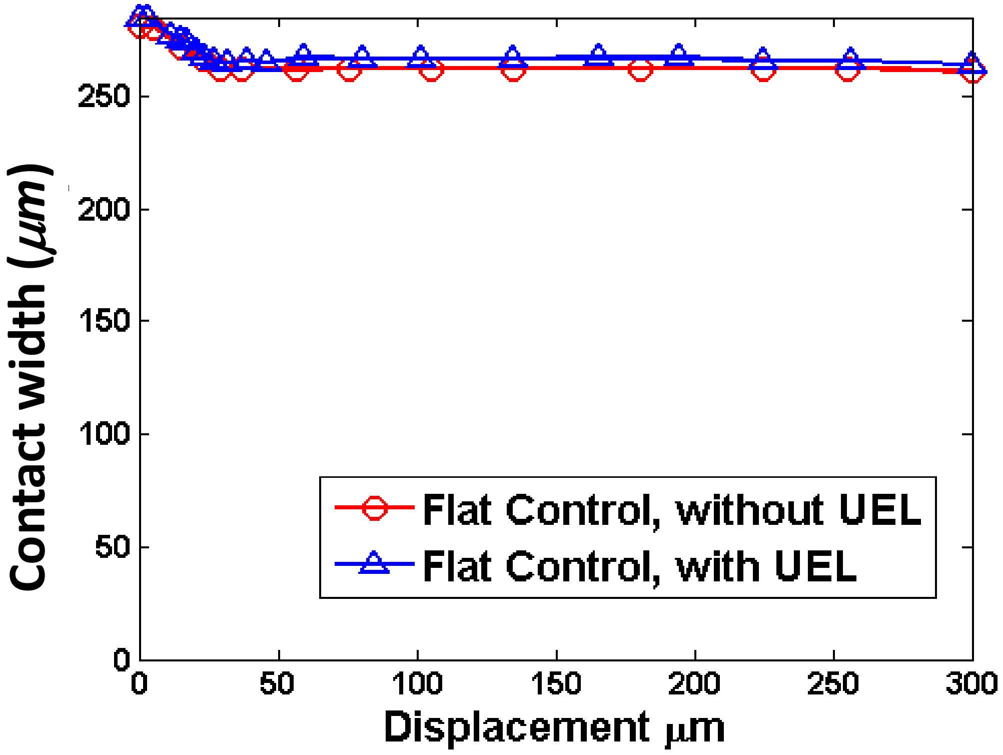

Figure S11. Comparison of contact diameter for Flat Control based on finite element simulation without and with adhesion (UEL is abbreviation for user element for adhesion).

SI.4. Enhanced Friction: Estimating the Contribution of Various Deformation Modes

In the main text of this manuscript, we introduced four mechanisms that potentially contribute to the measured adhesion in addition to the sliding friction of the indenter against the top surface. In the following, based on examination and analysis of videos of the deformation, we estimate the contribution to the enhanced friction from each of these mechanisms for the sample with largest spacing (D40S125). We estimate the various contributions to work and energy dissipation as the indenter moves relative to the sample by a distance, *S*. In a frame of reference attached to the material, the net action is equivalent to taking material of width *S* from far in front of the contact, through the deformation history, to a position far at the back of the contact. The external system performs work *FS* during this process. By equating this work to the sum of all dissipative processes that occur as a slice of material *S* in width is taken from the front of the contact to its rear, we can estimate *F*. In particular, we can examine which of the proposed mechanisms contributes significantly to the sliding friction force. Fig. S12 shows the schematic of the structure deformation. The typical measured value of *F* in this experiment is 45 mN for S125 and 12 mN for the control. Other parameters are:

Fibril width: c = 10 μm.
Distance S=125 μm.
Terminal film thickness: h =10 μm.
PDMS properties: E is the Young's modulus (2.85e+6)
Ridge height: D= 40 μm.
Contact width: a=300 μm.
Friction: τ (constant frictional stress, 200 kPa)

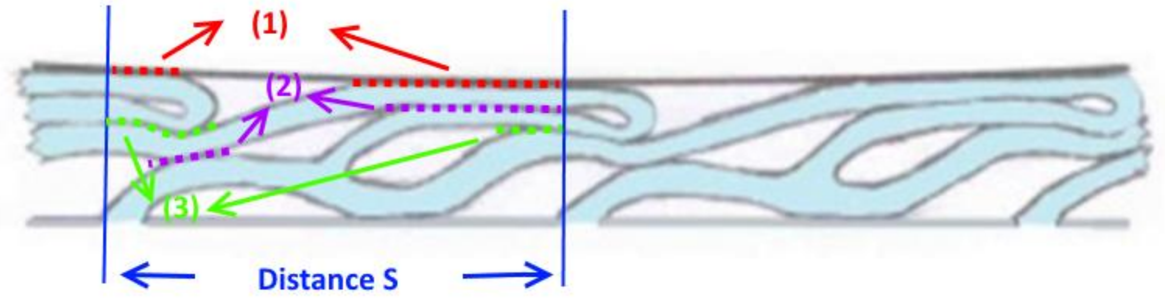

Figure S12. Schematic of deformation of ridges and top film for sample D40S125. The dotted lines show the contact pair: indenter to top film (1), bottom surface of top film to itself and ridge (2), and top surface of top film to itself due to folding (3).

The contact width, *a*, is estimated based on the contact region (in video) and the consideration that gaps exist between indenter and top film, which reduces its value. Fig. S13 shows the contact width taken for sample D40S125 during sliding. There are two types of regions we need to distinguish. The first is the overall region over which significant deformation of the substrate occurs. This is significantly larger than the actual area of contact and this region does increase significantly as the ridges bend. There is a second, smaller region enclosed within the larger overall one. Within this region the ridges have bent and collapsed onto the substrate and the top

film is bent and folded. It is the area of this second region that we wish to measure in order to estimate the contribution of various terms such as internal friction to the overall sliding friction force. The inner region in Fig. S13 is identifiable by the fact that collapse of the ridge onto the substrate makes that region distinctly darker, as shown by the dashed circle in Fig. S13. We estimate the contact diameter as that of a circle that circumscribes the inner region.

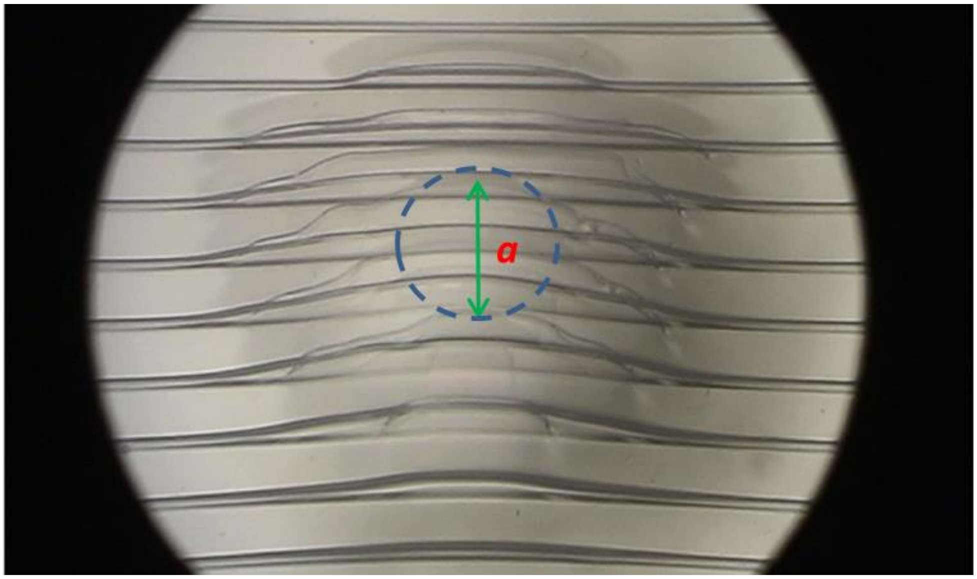

Figure S13. Schematic of contact width, *a*, for sample D40S125 during sliding.

1. Stretching of ridges:

During sliding, the ridges stretch considerably. Analysis of the videos reveals that the ridges are stretched to about 170% of their original length (height) and are released suddenly. We presume that the stretching energy is released unstably and is therefore lost when the ridges come out of contact. After being stretched, the ridge is prolonged from *l* to *l'*, and the energy stored per unit out-of-plane length can be estimated from the following equation (based on a Neo-Hookean material description) in which λ is the ratio of *l'* to *l*.

$$U_1 = \frac{1}{6} cDE\left(\lambda^2 + \frac{1}{\lambda^2} - 2\right) \qquad \text{Equation (S1)}$$

The contribution to the average friction force is expressed in Equation S2.

$$F_1 = aU_1 / w \qquad \text{Equation (S2)}$$

which evaluates to about 0.57 mN. Hence the contribution to friction from this mechanism is negligible.

2. Stretching of roof film:

The top film experiences considerably more severe stretching, also followed by its unsteady release. The top film is folded and both sections are stretched. Based on the video, the initial length of the roof film, *S*, is stretched to a length of 3 *S*. The energy, U_2, stored in top film

can be calculated from eq. S1 with c replaced by h, l replaced by w and λ of value 3. Supposing the top film is stretched evenly, the contribution of stretching top film to average friction force is

$$F_2 = \frac{h}{6} aE\left(\lambda^2 + \frac{1}{\lambda^2} - 2\right) \qquad \text{Equation (S3)}$$

which evaluates to about 10 mN, and so this mechanism contributes quite significantly to friction.

3. Internal friction and external friction

Analysis of the video reveals that the ridges are stretched to about 1.7 times of their original length, and top film (between two adjacent ridges) is stretched to about 3 times of its original length. The internal friction works when ridges and top film get stretched, which means the longest working distance of internal friction is $2w + 0.7D$. In Fig. S12 the contact pairs are shown by dotted lines of different colors, in which the gap is overstated compared to experimental observation.

The contribution of the indenter to top film contact pair (Fig. S12, red dotted line) to total friction per unit length out-of-plane can be calculated from Eq. S5.

$$F_4 = \tau \times a^2 \qquad \text{Equation (S5)}$$

The contribution originating from the bottom surface of top film sliding on ridge, itself and substrate (Fig. S12, purple dotted line) can be calculated from Eq. S6.

$$F_5 = a\frac{\int_0^{w+D} \tau \times \frac{x}{w+D} \times (2w+0.7D)\, dx}{w} = a\frac{\tau(w+D)\times(2w+0.7D)}{2w} \qquad \text{Equation (S6)}$$

The contribution originating from the top surface of the top film sliding on itself (Fig. S12, green dotted line) can be achieved from Eq. S7 in which τ_{max} is w.

$$F_6 = a\frac{\int_0^{x_{max}} \tau \times x \times dx}{w} = a\frac{\tau w}{2} \qquad \text{Equation (S7)}$$

The sum of F4, F5, and F6 evaluates to about 32 mN, so this is clearly the dominant mechanism.

4. Adhesion hysteresis

The energy difference for creating and breaking contact is also one contribution to the friction. Based on the videos including side-view analysis, due to the folding of top film and falling down of ridges, the contact surface pairs are: indenter to top film surface, top film to itself due to folding, top film to ridges, ridges to substrate and top film to substrate. The contribution to friction per unit length is denoted as F_3 which can be calculated from the following equation in which Δ_w is the difference between opening and closing interfaces and equals to 100 J/m^2.

$$F_3 = \mathrm{a}\ \Delta_w \times w \times 4\ /\ w \qquad \text{Equation (S8)}$$

This has a nearly negligible contribution of about 0.12 mN.

SI.5. Condition for Collapse of Terminal Film Onto Substrate

Here we estimate the condition for existence of a metastable state in which the film can exist in a collapsed state, adhered to the substrate between two ridges. We model the terminal film as a membrane under tension *T*. Based on peeling mechanics[3], tension and energy release rate are related by

$$W_{ad} = T(1-\cos\theta) \qquad \text{Equation (S9)}$$

where θ is the contact angle. The membrane is a straight line and its stretch is

$$\lambda = \frac{\sqrt{S^2/4+D^2}}{S/2} \approx 1 + \frac{2D^2}{S^2} \qquad \text{Equation (S10)}$$

For a neo-Hookean membrane,

$$\mathrm{T} = \mu h\left(\lambda - \frac{1}{\lambda^3}\right) \approx \mu h\left(1 + \frac{2D^2}{S^2} - \left[1 + \frac{2D^2}{S^2}\right]^{-3}\right) \approx 8\mu h\, D^2/S^2 \qquad \text{Equation (S11)}$$

Since

$$\cos\theta = S/\sqrt{S^2+4D^2} \qquad \text{Equation (S12)}$$

We have

$$W_{ad} \approx 8\mu h\, D^2/S^2(1-\cos\theta) \approx \frac{8\mu h D^2}{S^2}\left(1 - \left[1 + \frac{4D^2}{S^2}\right]^{1/2}\right) = \frac{16\mu h D^4}{S^4} \qquad \text{Equation (S13)}$$

Thus, the condition for collapse is

$$W_{ad} \geq \frac{16\mu h D^4}{S^4} \Rightarrow \frac{W_{ad} S^4}{16\mu h D^4} \geq 1 \qquad \text{Equation (S14)}$$

SI.6. Condition for buckling of terminal film

The following is a simple model for the condition that decides, during sliding on a film-terminated ridge-channel structure, whether the surface will start to fold resulting ultimately in high sliding friction, or whether it will just go into regular contact with either the same sliding friction as a control if no voids open (old design with rounded corners) or reduced friction if voids open up at the interface. Figure S14 shows a schematic drawing of an indenter sliding on the film-terminated ridge structure.

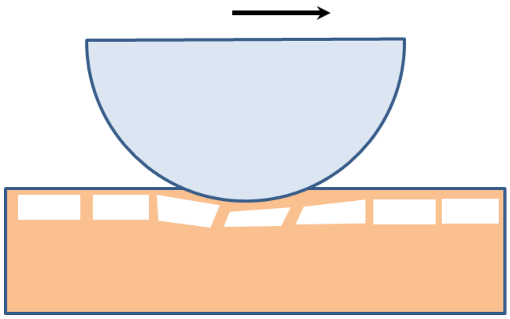

Figure S14. Schematic drawing of an indenter sliding on the film-terminated ridge structure.

Figure 3 (main manuscript) shows a plane strain finite element simulation of rigid indenter sliding on a film-terminated ridge structure. There is loss of contact underneath the indenter. However, our hypothesis is that the critical event corresponding to transition to high sliding friction is buckling of the film just before it enters the contact region.

We develop a model for this condition in two parts. In the first part we calculate the force on the portion of the film about to enter the contact zone. In the second part we ask: is this force sufficient to buckle the film entering the contact zone?

Definitions:
Fibril width: *c*
Periodic spacing: *w*=*S*
Terminal film thickness: *h*.
PDMS properties: *E** is the plane strain modulus; no adhesion in the model; material is incompressible.
Ridge height: *D*
Contact width: *a*
Friction: τ (constant frictional stress)

Part 1. Force at the contact edge

Figure S15(a) shows a schematic drawing of the terminal film of our structure during sliding under an indenter. Also shown in the figure are the horizontal forces. We assume that the combined action of the ridges and the elastic substrate below it, can be represented as a distributed spring foundation that provides a resisting force (per unit width out-of-plane) to the terminal film of $-k_f u$. The negative sign indicates that the force resists shear deformation.

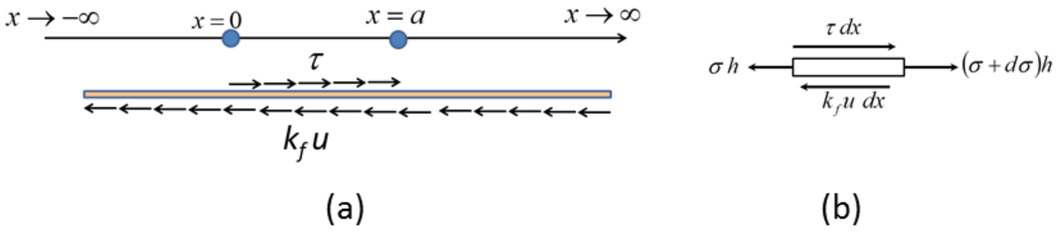

Figure S15 (a) Schematic drawing of the terminal film showing the horizontal forces acting on it. The terminal film is separated from the ridges and the indenter. The force provided by the indenter is represented by constant frictional stress τ acting on the contact region. The shear force acting on the film from the ridges is proportional to the local displacement u of the film since this is also the displacement of the top of the ridge with respect to its bottom. (b) Balance of horizontal forces on a small element of the film of length *dx*.

The shear spring constant k_f, with units of N/m^3, is related to geometric and material properties using simple beam theory:

$$k_f = \frac{3E^* I_p}{wDl^3} = \frac{E^* c^3}{4wDl^3} \qquad \text{Equation (S15)}$$

Balance of forces in the horizontal direction (Figure S15(b)) requires

$$h\frac{d\sigma}{dx} - k_c u = -\tau \qquad \text{Equation (S16)}$$

Combining with the stress-strain relation

$$\sigma = E^* \varepsilon = E^* \frac{du}{dx}, \qquad \text{Equation (S17)}$$

equation (S16) becomes

$$\frac{d^2u}{dx^2} - \frac{k_f}{E^* h} u = -\frac{\tau}{E^* h} \qquad \text{Equation (S18)}$$

The domain is in three parts,

$$\begin{aligned} I &\to x < 0; \\ II &\to 0 \le x < a; \\ III &\to a \le x \end{aligned} \qquad \text{Equation (S19)}$$

and $\tau = 0$ except in region *II*. The solution in region *II* is

$$u_{II} = C_1 \sinh(\alpha x) + C_2 \cosh(\alpha x) + \frac{\tau}{k_f}$$

$$\alpha = \sqrt{k_f / (E^* h)} \qquad \text{Equation (S20)}$$

The solution in region *I* is

$$u_I = C_3 \exp(\alpha x) \qquad \text{Equation (S21)}$$

where we have dropped the exponential term $u_I \sim \exp(-\alpha x)$ since the solution must vanish as $x \to -\infty$. Similarly, the solution in region *III* is

$$u_{III} = C_4 \exp(-\alpha x) \qquad \text{Equation (S22)}$$

The solution, equations (S20, S21, S22) contains four unknown constants and we impose four conditions to determine these: continuity of displacement and stress at the boundary between regions *I* and *II*, and between *II* and *III*.

$$u_I(0) = u_{II}(0)$$
$$u'_I(0) = u'_{II}(0)$$
$$u_{II}(a) = u_{III}(a)$$
$$u'_{II}(a) = u'_{III}(a)$$

Equation (S23a-d)

Equations (S23a-d) give, respectively,

$$C_3 = C_2 + \frac{\tau}{k_f}$$
$$C_1 = C_3$$
$$C_1 \sinh(\overline{\alpha}) + C_2 \cosh(\overline{\alpha}) + \frac{\tau}{k_f} = C_4 \exp(-\overline{\alpha});$$
$$C_1 \cosh(\overline{\alpha}) + C_2 \sinh(\overline{\alpha}) = -C_4 \exp(\overline{\alpha})$$
$$\overline{\alpha} = \alpha a = \sqrt{k_f a^2 / (E^* h)}$$

Equation (S24a-d)

Eliminating C_3 and C_2 using equations (S24a,b), this reduces to

$$C_1 \sinh(\overline{\alpha}) + \left(C_1 - \frac{\tau}{k_f} \right) \cosh(\overline{\alpha}) + \frac{\tau}{k_f} = C_4 \exp(-\overline{\alpha});$$
$$C_1 \cosh(\overline{\alpha}) + \left(C_1 - \frac{\tau}{k_f} \right) \sinh(\overline{\alpha}) = -C_4 \exp(\overline{\alpha})$$

Equation (S25a-b)

Eliminate C_4 by adding S25(a,b), then solve the remaining equation to get

$$C_1 = \frac{\tau}{2k_f} \left(1 - \frac{1}{\sinh(\overline{\alpha}) + \cosh(\overline{\alpha})} \right)$$ Equation (S26)

Putting (S26) into S25 (b) and simplifying, we find

$$C_4 = \frac{\tau}{2k_f} (\exp(\overline{\alpha}) - 1)$$ Equation (S27)

We are interested in calculating the force P (per unit length out of plane) at $x=a$, which is

$$P = hE^* \left.\frac{du}{dx}\right|_{x=a} = -hE^* C_4 \alpha \exp(-\overline{\alpha})$$
$$= -hE^* \frac{\tau}{2ak_f} (1 - \exp(-\overline{\alpha}))\overline{\alpha}$$

Equation (S28)

Combining 24(d) and (S15) we get

$$\overline{\alpha} = \alpha a = \frac{1}{2} \sqrt{\frac{c^3}{D^3} \frac{a^2}{wh}}$$ Equation (S29)

Using (S29) and (S15) in (S29), we get

$$P = 2 \frac{h}{a} \frac{D^3}{c^3} \tau w (1 - \exp(-\overline{\alpha}))\overline{\alpha}$$ Equation (S30)

Part 2: Buckling of the film just ahead of the leading contact line

We are interesting in the condition for buckling of the plane strain film just ahead of the advancing contact line. For a more accurate analysis, we should consider a long film with many tethers to the substrate, accounting for the torsional rigidity of each one. This can be done and produces a transcendental equation that will in any case need to be solved numerically. Since we wish to extract the main scaling behavior, we opt for a simpler description in which we consider the film as a plane strain beam with simple boundary conditions. In that case, the buckling load (force per unit length) is

$$P = \beta \frac{E^* I_f}{w^2}; \quad \beta = \begin{cases} \pi^2 & ss-ss \\ 9\pi^2/4 & ss-c \\ 4\pi^2 & c-c \end{cases} \qquad \text{Equation (S31)}$$

Here, I_f is the moment of inertia of the cross-section of the film. That is,

$$P = \beta \frac{E^* h^3}{12 w^2}, \qquad \text{Equation (S32)}$$

Combining the two parts

Combining the results of part 1 (S30) and part 2 (S31), we get the condition:

$$\left(1-\exp\left(-\bar{\alpha}\right)\right)\bar{\alpha} > \frac{\beta}{24}\frac{a}{w}\left(\frac{h}{w}\right)^2\left(\frac{c}{D}\right)^3\frac{E^*}{\tau}; \quad \bar{\alpha} = \alpha a = \frac{1}{2}\sqrt{\frac{c^3}{D^3}\frac{a^2}{wh}} \qquad \text{Equation (S33)}$$

It turns out that we are closer to the limit of large $\bar{\alpha}$, in which case the LHS of (S33) itself is $\bar{\alpha}$. Then, the condition simplifies to

$$\frac{12}{\beta}\frac{\tau}{E^*}\left(\frac{w}{h}\right)^{5/2}\left(\frac{D}{c}\right)^{3/2} > 1; \qquad \text{Equation (S34)}$$

Example: Let us set h = 10.0 μm; c = 10 μm; D = 40 μm; E* = 3.7 MPa; β= 4 Pi^2, *t* = 50 kPa (about 1/3 of the true frictional stress because of loss of contact). We find that (S34) is satisfied for separation > 50 μm.

SI.7. Information for supplementary video files

The description for each video file is shown in Table S1, describing the condition of carrying out experiment and which sample it corresponds to. To capture the deformation more accurately, videos were taken from both the bottom and side. In the description of videos, indenter moving speed, moving direction, and normal load condition are provided.

Video #	File Name	Legend
1	D40S20_Sideview_Speed5_1.mp4	Side view video: Sample D40S20; indenter is moving orthogonal to ridges at speed 5 μm/sec.
2	D40S65_move_Speed5_10g_1.mp4	Video of sample D40S65; indenter is moving orthogonal to ridges at speed 5 μm/sec under normal load of 1 mN.
3	D40S65_movealong_Speed5_10g_1.mp4	Video of sample D40S65; indenter is moving along ridges at speed 5 μm/sec under normal load of 1 mN.
4	D40S125_move_Speed5_10g_1.mp4	Video of sample D40S125; indenter is moving orthogonal to ridges at speed 5 μm/sec under normal load of 1 mN.
5	D40S125_move_Speed5_10g_2.mp4	Video of sample D40S125; indenter is moving orthogonal to ridges at speed 5 μm/sec under normal load of 1 mN.
6	D40S125_movealong_Speed5_1.mp4	Video of sample D40S125; indenter is moving along ridges at speed 5 μm/sec under normal load of 1 mN.
7	D40S125_Sideview_Speed5_1.mp4	Side view video of sample D40S125; indenter is moving orthogonal to ridges at speed 5 μm/sec.
8	D40S125_Sideview_Speed5_2.mp4	Side view video of sample D40S125; indenter is moving orthogonal to ridges at speed 5 μm/sec.

Table S1. Description of each supplementary video file.

References

1. Shen, L., N.J. Glassmaker, A. Jagota, and C.-Y. Hui, *Strongly enhanced static friction using a film-terminated fibrillar interface.* Soft Matter, 2008. **4**(3): p. 618-625.
2. Rahul-Kumar, P., A. Jagota, S. Bennison, S. Saigal, and S. Muralidhar, *Polymer interfacial fracture simulations using cohesive elements.* Acta Mater., 1999. **47**(15): p. 4161-4169.
3. Kendall, K., *Thin-film peeling-the elastic term.* J. Phys. D: Appl. Phys., 1975. **8**(13): p. 1449.
